# Influence of Mass Media on Italian Web Users During the COVID-19 Pandemic: Infodemiological Analysis

**DOI:** 10.2196/32233

**Published:** 2021-10-18

**Authors:** Alessandro Rovetta, Lucia Castaldo

**Affiliations:** 1 Research and Disclosure Division Mensana srls Brescia Italy

**Keywords:** COVID-19, Google Trends, infodemiology, infoveillance, infodemic, media coverage, mass media influence, mass media, social media

## Abstract

**Background:**

Concurrently with the COVID-19 pandemic, the world has been facing a growing infodemic, which has caused severe damage to economic and health systems and has often compromised the effectiveness of infection containment regulations. Although this infodemic has spread mainly through social media, there are numerous occasions on which mass media outlets have shared dangerous information, giving resonance to statements without a scientific basis. For these reasons, infoveillance and infodemiology methods are increasingly exploited to monitor information traffic on the web and make epidemiological predictions.

**Objective:**

The purpose of this paper is to estimate the impact of Italian mass media on users’ web searches to understand the role of press and television channels in both the infodemic and the interest of Italian netizens in COVID-19.

**Methods:**

We collected the headlines published from January 2020 to March 2021 containing specific COVID-19–related keywords published on PubMed, Google, the Italian Ministry of Health website, and the most-read newspapers in Italy. We evaluated the percentages of infodemic terms on these platforms. Through Google Trends, we searched for cross-correlations between newspaper headlines and COVID-19–related web searches. Finally, we analyzed the web interest in infodemic content posted on YouTube.

**Results:**

During the first wave of COVID-19, the Italian press preferred to draw on infodemic terms (rate of adoption: 1.6%-6.3%) and moderately infodemic terms (rate of adoption: 88%-94%), while scientific sources favored the correct names (rate of adoption: 65%-88%). The correlational analysis showed that the press heavily influenced users in adopting terms to identify the novel coronavirus (cross-correlations of ≥0.74 to ≤0.89, *P* value <.001; maximum lag=1 day). The use of scientific denominations by the press reached acceptable values only during the third wave (approximately 80%, except for the television services Rai and Mediaset). Web queries about COVID-19 symptoms also appeared to be influenced by the press (best average correlation=0.92, *P*<.007). Furthermore, web users showed pronounced interest in YouTube videos of an infodemic nature. Finally, the press gave resonance to serious “fake news” on COVID-19, which caused pronounced spikes of interest from web users.

**Conclusions:**

Our results suggest that the Italian mass media have played a decisive role in spreading the COVID-19 infodemic and addressing netizens’ web interest, thus favoring the adoption of terms that are unsuitable for identifying COVID-19. Therefore, the directors of news channels and newspapers should be more cautious, and government dissemination agencies should exert more control over such news stories.

## Introduction

### Background

The COVID-19 pandemic has placed a strain on economies and health systems worldwide [1,2]. As of March 21, 2020, the death toll had reached approximately 2.71 million, and the trend continued to grow [3]. In addition to the disease, the world faced a growing infodemic that is capable of causing damage of equal severity [4]. The role of social and traditional media in spreading disinformation and misinformation has now been recognized in a substantial number of literature reports [5-10]. Indeed, numerous conspiracy hypotheses have circulated on the web regarding the imposition of lockdowns as tools of social control, the anthropogenic origin of the virus, its remedies and cures, and vaccines. This type of “fake news” can compromise compliance with antipandemic regulations and generate vaccine hesitancy [11-14]. Moreover, less obvious but equally incisive aspects exist within an infodemic. In particular, the assignment of scientific denominations to a new pathology represents a historically known public health issue [15]. For example, names such as “swine flu” and “Middle Eastern respiratory syndrome” have unwittingly contributed to fomenting racism and causing economic damage to specific industries [16]. The adoption of inappropriate nomenclature in the medical field has led to the administration of incorrect drugs; moreover, during the COVID-19 pandemic, the generic name “coronavirus” led the public to consult material relating to previous coronaviruses [17-19]. Some of the consequences are even unpredictable; for example, the name “coronavirus” has also been mistakenly associated with the Mexican firm Corona [16,20]. As shown by Su et al [21], mass media outlets such as newspapers and television news have played a relevant role in the proliferation of the COVID-19 infodemic, contributing to the resonance of stigmatizing names such as “Chinese virus.” In this scenario, Italy—the nation most affected by COVID-19 between the end of February and mid-March 2020 [2]—suffered damage from a severe infodemic. Although a large amount of misinformation has circulated on social networks, numerous television personalities, politicians, media outlets, and even scientists have contributed to spreading infodemic monikers, conspiracy theories (eg, the unproved laboratory origin of SARS-CoV-2), and misleading health-threatening information (eg, COVID-19 is like seasonal influenza) [9,10]. This climate of uncertainty immediately compromised trust in institutions (whose nonpharmacological interventions were often catalogued as exaggerated), fueled racism toward Chinese individuals residing in Italy, and altered the risk perception of the population. Hence, the Italian people have been fractionated between deniers, conspiracy theorists, reductionists, and people who are aware of the disease’s dangers.

### Countermeasures to the Infodemic

The COVID-19 situation requires an infoveillance system that is effective and efficient. The purpose of this system is not only to monitor information but address the community’s concerns [14]. Specifically, the discipline that deals with the determinants and distribution of health information and misinformation is called *infodemiology*, from the union of the terms “information” and “epidemiology.” Because the internet has accelerated and expanded the amount of information circulating globally, the primary purpose of infodemiology is to catalog disinformation (ie, the voluntary sharing of infodemic material) and misinformation (ie, the involuntary sharing of infodemic material) by seeking to identify the sources of and reasons for these phenomena [4,9,10]. During the COVID-19 pandemic, scientists have exploited different infodemiological approaches, ranging from machine learning to questionnaires [22,23]. However, these methods, although innovative and/or precise, need long implementation times before providing usable results. For this reason, a large part of the scientific community has conducted surveys through Google Trends, a tool developed by Google LLC that allows the user to obtain quantitative data on the web search volumes related to specific keywords in specific geographical areas during a preselected time period [24]. Google Trends dramatically simplifies and speeds data collection on netizens’ web interests, and it has been used to conduct medical, psychological, sociological, epidemiological, and economic investigations [25]. However, many authors have questioned its reliability because of the mass media’s influence on web searches [25-28]. Although this influence may jeopardize the adoption of Google Trends in many research areas, it remains suitable for conducting infodemiological investigations; in fact, if certain conditions are met (ie, high internet penetration, high Google use, and data set stability), the relative search volumes (RSVs) on Google Trends faithfully reflect the interests of a population [25]. Moreover, Sato et al [29] proposed a new method to evaluate, quantify, and eliminate the media disturbance in data sets.

### Research Questions

The internet represents a fast, user-friendly means to seek health-related information [9,10,30-32]. During a pandemic or any major health crisis, the need for quality web information is more pressing than ever; fear, anxiety, stress, and confusion due to the overabundance of often conflicting or dramatic news increases the consultation of web sources to seek remedies or reassurance [4,9,32-37]. As discussed above, the mass media plays an important role in managing an infodemic and conditioning web users’ behavior. Considering the vast infodemic that affected Italy during the COVID-19 crisis, this paper aims to answer the following research questions:

- R1: How much has the Italian media influenced Italian web users in the adoption of terms to identify COVID-19?
- R2: How much has the Italian media influenced the web interest in COVID-19?
- R3: Among the content disseminated by mass media channels, how much interest was aroused by infodemic news?

Alongside these questions, our analysis can provide further evidence on the reliability of Google Trends for studies other than infodemiological ones.

## Methods

### Design

To evaluate the impact of the media on Italian users’ web interest, we compared the use of specific keywords by the main Italian media with the RSVs of the same keywords, looking for significant and substantial (cross-)correlations, triggering events, and similarities in keyword adoption rates. Furthermore, we compared the use of scientific and infodemic names by the most-consulted newspapers and news channels with those of other platforms, such as PubMed, Google, and the website of the Italian Ministry of Health.

### Data Collection

Given the heterogeneity of the data collected, different methods were used for each type of source investigated. Specific keywords were selected in specific periods according to Table 1.

These keywords have been carefully selected through a previous literature analysis to represent the most adopted terms to identify SARS-CoV-2 and the consequent disease, COVID-19 [9,10]. We exploited the *infodemic scale* (I-scale) to assess the infodemicity of the terms examined: each moniker was assigned 1 to 2 points per category (ie, generic, misinformative, discriminatory, deviant, other specificities), ranging from 0 to 10. Based on the sum of the I-scale scores, the infodemic monikers were classified as follows: not infodemic (0), slightly infodemic (1), moderately infodemic (2-4), highly infodemic (5-8), and extremely infodemic (9-10). Further details on the use of the I-scale are given in [10].

Because search algorithms do not work in the same way on all databases, we provide below a summary schema of our collecting procedure.

**Table 1:** List of keywords searched on web-based platforms.

Investigated period	Period number	Searched keywords
Jan 1-Feb 13, 2020	1	*2019-ncov, novel coronavirus, coronavirus,chinese virus*, *chinese coronavirus*
Feb 11-May 18, 2020	2	*sars-cov-2*, *covid-related* (ie, *covid*, *covid 19*, *covid19*, *covid-19*), *novel coronavirus*, *coronavirus*
May 19, 2020-Mar 17, 2021	3	*sars-cov-2*, *covid-related* (ie, *covid*, *covid 19*, *covid19*, *covid-19*), *novel coronavirus*, *coronavirus*

#### PubMed

We searched PubMed [38] for each keyword individually and counted the results found. The *coronavirus* query also contained the results of the *novel coronavirus* query; therefore, calling the two set dimensions N and n, respectively, we calculated the number of results for the *novel coronavirus* query by subtracting n from N. We chose this source because it represents the largest medical database in the world.

#### Italian Ministry of Health

We searched the website of the Italian Ministry of Health [39] for each keyword individually and counted the results found. Specifically, we counted the number of official press releases per keyword. We chose this source because it is the official government website of the Italian Ministry of Health.

#### Google

We searched Google [40] for each keyword individually and counted the results found. In this case, we ensured that each webpage contained the specified keyword by including the latter in quotes (eg, “*coronavirus*”). We chose this source because Google represents the most adopted web engine in Italy [41].

#### Google Trends

We searched Google Trends for each keyword individually. We set the search category to “All categories” and downloaded the data as .csv files. We set the geographical region to “Italy” and analyzed the national web searches.

#### La Repubblica

We searched *La Repubblica* [42] for each keyword individually and counted the results found. Specifically, we retrieved the number of headlines starting on January 11, 2020, day by day (ie, from January 11 to 12, January 11 to 13, and so on). After that, we calculated the daily increment by subtracting the values of 2 consecutive days. We used the “advanced search” and “exact search” filters. We chose this source because it represents the second most read newspaper in Italy [43].

#### Il Corriere della Sera

We searched for each keyword on *Il Corriere della Sera* [44] individually and counted the results found. Specifically, we retrieved the number of headlines starting on January 11, 2020, day by day (ie, from January 11 to 12, January 11 to 13, and so on). After that, we calculated the daily increment by subtracting the values of 2 consecutive days. Because the *coronavirus* query also contained the results of the *novel coronavirus* query, calling the two set dimensions N and n, respectively, we calculated the number of results for the *novel coronavirus* query by subtracting n from N. We chose this source since it represents the most read newspaper in Italy [43].

#### TitoliGiornali (Other Newspapers)

We searched the website TitoliGiornali [45] for each keyword individually and counted the results found. Specifically, we counted the number of headlines per keyword. We chose this source because it includes the headlines of a wide variety of Italian newspapers.

#### Rai (Google News)

We searched Google [40] for each keyword individually, adding the term *rai* to each query, selected the item “Google News,” and counted the results found. We chose this source because Rai is the main Italian public television channel.

#### Mediaset (Google News)

We searched Google [40] for each keyword individually, adding the term *mediaset* to each query, selected the item “Google News,” and counted the results found. We chose this source because Mediaset is the main Italian private television channel.

#### YouTube

We searched YouTube [46] for each keyword individually and counted the results found. We added specific YouTube channel names to each query. We used the “sort by number of views” filter. We chose this source because it represents the largest video-sharing platform in the world.

### Statistical Analysis

#### Linear Regression

When data were normally distributed, the angular coefficient *m* of the interpolating line was calculated to evaluate the importance of a trend. Moreover, Pearson *R* and adjusted Pearson *R coefficients were calculated.

#### Mann-Kendall Test

To detect trends, after an initial graphical analysis, the Mann-Kendall test was used. Furthermore, the trend relevance was evaluated with the Sen slope (*SS*).

#### Mean Values

All average values are presented in the form of mean (standard error of the mean [SEM]). The variability of a data set was evaluated through the percentage standard deviation (SD%), calculated as the ratio between the standard deviation and the mean value multiplied by 100.

#### Normality Test

To verify the distributive normality of a data set, we used the Shapiro-Wilk test plus a graphical check of histograms and quantile-quantile diagrams.

#### Percentage Increases

We have indicated the percentage increases with the symbol Δ%.

#### Percentage Differences

We have indicated the percentage differences with the symbol δ%.

#### Pearson and Spearman Cross-Correlations

When the data sets were normally distributed, the Pearson correlation *R* was used; otherwise, the Spearman correlation *r* was used. The correlation strength was assessed independently of the *P* values. The lag range was set at a maximum of 50% of the data set size. The Olkin-Pratt method was used to calculate the weighted mean of a list of correlations (*ρ*).

#### *P* Values

*P* values were used as a continuous measure of the strength of evidence against the null hypothesis.

#### Seasonalities

To evaluate time-series seasonalities, we used a graphical check and the Time Series Analysis tool of the XLSTAT package (Addinsoft). In particular, we divided each signal into trend, seasonal, and random components. Finally, we calculated the autocorrelogram plot.

#### Welch *t* Test

When the data sets were found to be normally distributed, the Welch *t* test was used. Furthermore, when the size of the analyzed sets exceeded 30 elements, the central limit theorem was exploited to use the Welch *t* test even with data that were not normally distributed [47,48].

#### Software

Excel 2020 (Microsoft Corporation) was used for data analysis. In addition, the Real Statistics and XLSTAT packages were used.

## Results

### Media Influence on Italian Web Users

At the beginning of the COVID-19 pandemic, we found a substantial difference in the adoption of the scientific names “2019-nCoV” and “novel coronavirus” against the more infodemic term “coronavirus” between the most read Italian newspapers and sources such as Google and PubMed (Table 2).

Moreover, although the streaming video portals RaiPlay and MediasetPlay by Rai and Mediaset (the main Italian television broadcasters) did not show any results for the item *2019-ncov*, they produced several results for the queries *Chinese virus* and *Chinese coronavirus*. These websites also have special sections entitled “Coronavirus.” According to Google results from January 1 to February 13, 2020, the same websites used the term *coronavirus* in 93.8% of article titles, compared to 6.3% for *2019-ncov* and 0% for *novel coronavirus*. In this context, the Italian web users immediately preferred the term *coronavirus* (mean RSV=14) over all the others (∀RSV<1), showing values comparable to those of the most relevant newspapers and television broadcasters in Italy. Moreover, *Chinese virus* and *Chinese coronavirus* were the second most commonly used keywords during this phase (mean RSV=22 vs mean ∀RSV≤1, excluding the query *coronavirus*), showing further similarities with the Italian media.

**Figure 1:**
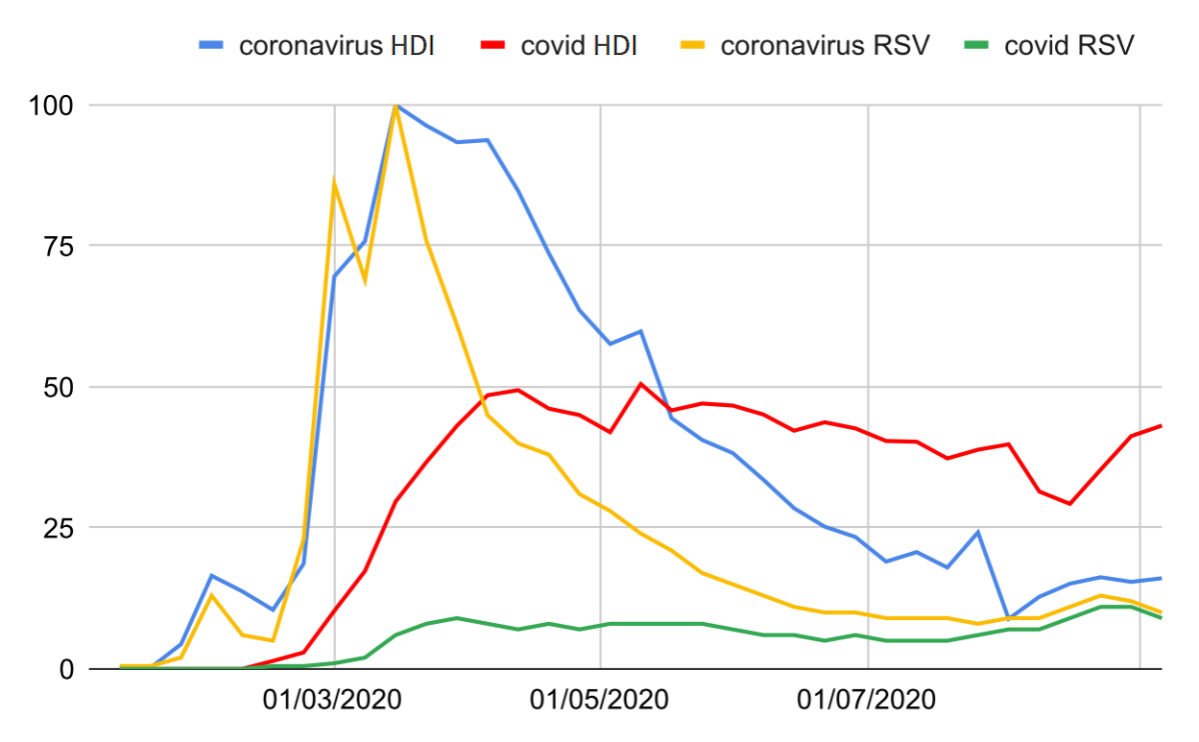
Comparison of the weekly RSVs of the keywords *coronavirus* (yellow) and *covid* (green) with the number of times the terms were adopted by the newspaper *La Repubblica* (blue and red, respectively) from January 1 to September 6, 2020. All values were normalized to 100. HDI: headlines daily increase; RSV: relative search volume.

Considering the keyword *coronavirus*, the weekly-cumulative RSV showed a pronounced correlation with its weekly cumulative use by the newspaper *La Repubblica* (Figure 1). The best overall cross-correlation was *r*=0.89 (95% CI 0.80-0.95), *P*<.001; lag=0 weeks.

**Table 2:** Rates of adoption of COVID-19–related terms at the start of the pandemic (January 1 to February 13, 2020).

Source	Rate of adoption (%)			
	2019-ncov (infodemic scale: 0)	Novel coronavirus (infodemic scale: 1)	Coronavirus (infodemic scale: 4)	Chinese (corona)virus (infodemic scale: 9)
PubMed	42.7	45.2	12.1	<0.1
Italian Ministry of Health	11.8	52.9	35.3	0.0
Google	29.3	25.9	27.6	17.2
*La Repubblica*	0.2	5.3	92.9	1.6
*Il Corriere della Sera*	1.3	4.7	87.7	6.3
Other newspapers^a^	0.5	0.1	93.9	5.4
Rai (Google News)	1.6	0	96.8	1.6
Mediaset (Google News)	0	0	97.8	2.2

^a^This item includes *Il Sole 24 Ore*, *Il Fatto Quotidiano*, *Il Giornale*, and *La Stampa*.

**Figure 2:**
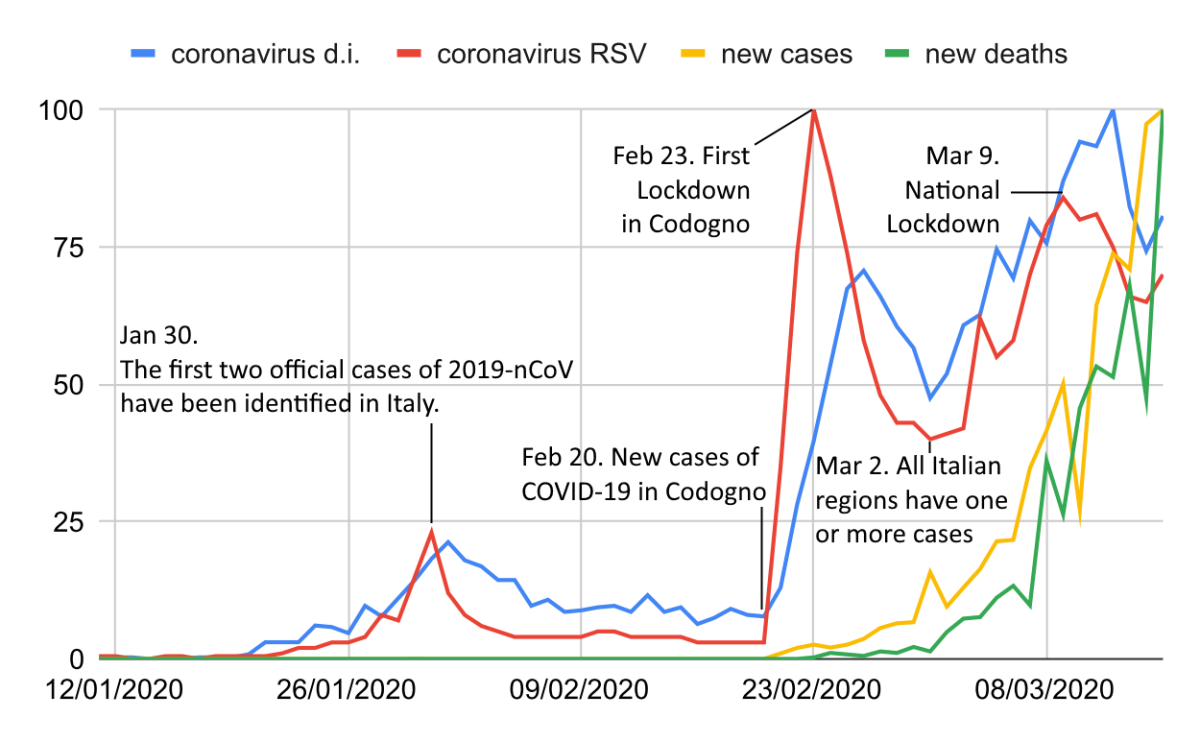
Comparison between the daily RSVs (red) of the keyword *coronavirus*, new COVID-19 cases, and COVID-19 deaths, and the number of times they were adopted by the newspaper *La Repubblica* (blue) from January 11 to March 15, 2020. All values are normalized to 100. The black lines represent the beginning or the end of a trend. d.i.: daily increase; RSV: relative search volume.

In the period from the week of January 13 to 19 to that of the peak during March 9 to 15, the average percentage discrepancy between all data pairs was significantly smaller than in the following months up to August (δ%=51.4, *t*=3.8). By restricting the analysis to the period from January 19 to March 12, 2020 (initial phase), to observe daily cumulative values (Figure 2), 5 trends with significant correlates were observed (Multimedia Appendix 1). The unlagged initial phase correlation was *r*=0.93 (95% CI 0.88-0.96), *P*<.001, while the optimum cross-correlation was *r*=0.94 (95% CI 0.91-0.97), *P*<.001, lag=1 day. From March 13 onward, a decreasing trend was observed for both variables until the week of August 2 to 8, 2020.

For *Il Corriere della Sera*, the results were slightly different: first, the percentage discrepancies between the RSV and daily increase in headlines for *coronavirus* were statistically confident (δ%=7.6, *t*=0.5); second, among the 6 investigated correlations, some were weak (Multimedia Appendix 1). However, the optimum initial phase correlation was *r*=0.84 (95% CI 0.71-0.92), *P*<.001, lag=0 days, and the best overall cross-correlation was *r*=0.85 (95% CI 0.73-0.92), *P*<.001, lag=0 weeks.

After the introduction of the scientific names SARS-CoV-2 and COVID-19, the use of the moderately infodemic and noninfodemic or slightly infodemic names partially changed compared to the previous period (Table 3). However, even in this case, all the media are characterized by greater use of the generic name “coronavirus.”

In the period from February 11 to May 18, 2020, the terms *SARS-CoV-2* and *COVID-related* were used differently by Italian users (∀RSV<1 vs mean RSV=64, respectively). The term *COVID-related* was used less frequently than *coronavirus* until the second week of September (Δ%=404, *t*=5.0). Before the second week of May, the weekly adoption of the term *COVID-related* by *La Repubblica* was significantly lower than that of the term *coronavirus* (∀∆% ∈ [18;642]). Furthermore, the percentage discrepancies between the RSV and daily increase in headlines for *COVID-related* were substantial (mean Δ%=509, SEM 32, in the whole investigated period). Nonetheless, correlations were found from the week of February 10 to 16 to that of July 20 to 26 (optimum overall cross-correlation: *r*=0.74, 95% CI 0.49-0.88; *P*<.001; lag=0 weeks). Finally, among all the COVID-19–related terms, *covid* was the one most exploited by users despite the more appropriate and specific *covid-19* (mean RSV=47 vs mean RSV=2). The results for *Il Corriere della Sera* were similar (mean RSV ∆%=773, SEM 69; optimum overall cross-correlation: *r*=0.73, 95% CI 0.48-0.88; *P*<.001; lag=0 weeks).

**Table 3:** Rates of adoption of COVID-19-related terms during the lockdown (February 11 to May 18, 2020).

Source	Rate of adoption (%)			
	SARS-CoV-2 (infodemic scale: 0)	COVID (and related) (infodemic scale: 1)	Novel coronavirus (infodemic scale: 1)	Coronavirus (infodemic scale: 4)
PubMed	27.5	38.6	28.8	5.1
Italian Ministry of Health	1.3	78.2	9.0	11.5
Google	24.3	28.4	25.7	21.6
*La Repubblica*	0.6	33.2	1.3	64.8
*Il Corriere della Sera*	1.2	44.0	1.0	53.8
Other newspapers^a^	1.6	39.9	1.4	57.1
Rai (Google News)	0.0	15.4	0.6	84.0
Mediaset (Google News)	0.0	7.0	0.7	92.3

^a^This item includes *Il Sole 24 Ore*, *Il Fatto Quotidiano*, *Il Giornale*, and *La Stampa*.

### Evidence Supporting Causation

Noncausal correlations could be due to stochastic phenomena (hypothesis a) or other triggering phenomena (spurious correlations, hypothesis b). We found up to 6 significant consecutive trends (Mann-Kendall mean *P*=.007, SEM .006; ∀P<.03) and as many strong correlations between RSVs and newspaper headlines (Multimedia Appendix 1). Let us consider hypothesis a, thus supposing that the correlations found are due to chance. A realistic estimate was obtained by generating random values using the random number generator provided by Haahr [49]: in particular, by generating 50 data series composed of 6 numbers and looking for 1225 Pearson and Spearman correlations between them, significant correlations (ie, close to the threshold *α*=.05) were obtained in approximately 6.5% of cases; consequently, the probability of obtaining 6 consecutive significant correlations was (6.5/100)^6^ < 10^-6^. Furthermore, the hypothesis investigated was targeted, avoiding the problem of the look-elsewhere effect. Indeed, this targeting was based on data from this paper (eg, the rate of adoption of COVID-19–related terms) and previous works. Hence, these correlations can be considered to be not due to chance.

**Figure 3:**
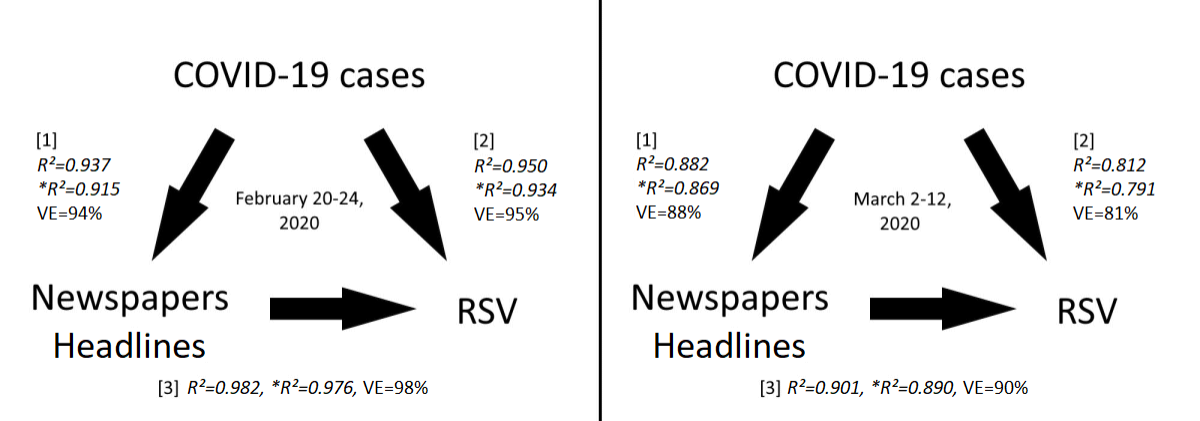
Causal implications scheme and linear regression results. RSV: relative search volume; VE: variability explained.

Now, suppose that both RSVs and newspaper headlines were causally influenced by a third quantity x. This quantity must have been linked to the COVID-19 epidemic. For this reason, we started searching for correlations between RSV and COVID-19 cases and deaths (C19). As shown in Figure 2, the trend of C19 shows a monotonic increase, while that of the web searches is not monotonic; this precludes the occurrence of global correlations. Thus, we start assuming the existence of local cross-correlations of type *COVID-19 symptoms → RSV → C19* (ie, such that web searches are predictive of C19). Based on the incubation and swab analysis periods, the average lag between web searches and symptom identification should be around 6 to 9 days [50-52]; nevertheless, Figure 2 shows that the first RSV trend precedes the growth of COVID-19 cases by 1 month. In contrast, suppose now a causal cross-correlation of type *C19 → RSV*. Considering the time-lapse from February 20 to March 13, 2020, we found 3 distinct groups of correlations (2 positives and 1 negative, with 0.83≤|*r*|≤0.98, ∀*P*≤.02) (Figure 2). After that, although COVID-19 cases continued to rise until April, both the RSV and “coronavirus” newspaper headlines started to decline. Alongside this, we performed multiple regression analyses between the quantities *COVID-19 cases*, *newspaper headlines*, and *RSV* following the scheme shown in Figure 3. These results also suggest a greater influence of newspapers on RSV, although there is a marked dependence among all the variables. By restricting the data sets to the periods where a positive correlation was found and improperly assuming independence between the normalized variables *COVID-19 cases* (X1) *and newspaper headlines* (X2), we calculated the least squares multiple regression plan for the *RSV* (y), obtaining y = 16X1 + 1.7X2 – 17 and y = 0.05X1 + X2 – 15.

Although it is clear that in some cases, both COVID-19 cases and newspapers contributed to conditioning Italian web users, COVID-19 cases had a more temporary and discontinuous effect than newspaper headlines. At any rate, it must be considered that even this type of news is disseminated by disclosure sources, which can be official government sources (ie, the Italian Ministry of Health, hypothesis I), private websites, blogs, and social networks (hypothesis II), or media (hypothesis III). Hypothesis I assumes that all citizens, once the COVID-19 issue became known, consulted official sources. Considering that the official Italian Government Printing Offices used, in the vast majority of cases, fewer infodemic names (83.5%), and the amount of news they reported was limited and not correlated with RSV (eg, from January 1 to March 12, the Ministry of Health produced 91 articles, compared to tens of thousands by media outlets), we can exclude this hypothesis. Hypothesis II assumes that the RSV was influenced by unofficial sources not directly related to press and newscasts. Because press and newscasts have their own independent sources and we already excluded every random correlation as well as any global correlations with COVID-19 cases, we can only suppose a causal global process of the type *media → private websites, blogs, social networks → RSV*, which confirms the fundamental role of media. Therefore, the only hypothesis with empirical evidence is that media, such as press and newscasts, have predominantly determined the web search trends and the terms used to conduct these searches (ie, Hypothesis III). Further support for this conclusion is provided by the fact that RSV peaks are always associated with highly impacting media events, such as lockdowns and outbreaks (Figure 2). Finally, web searches related to COVID-19 symptoms followed a very similar trend (Multimedia Appendix 1).

### COVID-19 Web Interest During the Second Wave

The *coronavirus + covid* query had a near-stationary RSV between early June and early August (Mann-Kendall *P*=.14, SS=–0.09, SD%=8.3). This was much less pronounced than during the first wave *(∆%*=–71.4*, t*=–14.2) due to the sharp decrease in COVID-19 cases and the easing of containment measures. From August 2020 to March 17, 2021, web interest in COVID-19 remained significantly lower than that during the first lockdown (∆%=–57.8, *t*=–4.2), thus showing spikes of RSV at the start of the second wave (RSV_max_=23, SEM 0.4, August 2020), a steep rise of cases from October 1, 2020, until around mid-November (RSV_max_=35, SEM 0.7) and mid-February, anticipating the increase in infections at the end of February by approximately 10 days (RSV_max_=23, SEM 0.5). The use of the term *covid* surpassed that of the more generic and infodemic term *coronavirus* (59% vs 41% from September 2020 to March 2021, respectively). In contrast, the more technical term *SARS-CoV-2* was not adopted by web users (∀RSV<1). On this occasion, as far as the adoption of COVID-19–related terms was concerned, strong differences were noticed between newspapers and other sources, including Rai and Mediaset (Table 4).

Although the use of COVID-19–related terms by newspapers exceeded that of the generic term *coronavirus* in May 2020, web users showed a delay of 4 months before doing the same. Finally, web users’ interest in COVID-19 decreased by approximately 20% compared to that during the first wave.

**Table 4:** Rates of adoption of COVID-19–related terms during the lockdown (May 19, 2020, to March 17, 2021).

Source	Rate of adoption (%)			
	SARS-CoV-2 (infodemic scale: 0)	COVID (and related) (infodemic scale: 1)	Novel coronavirus (infodemic scale: 1)	Coronavirus (infodemic scale: 4)
PubMed	26.6	42.9	27.6	2.8
Italian Ministry of Health	0.9	90.9	1.8	6.4
Google	25.7	28.6	22.9	22.9
*La Repubblica*	0.9	76.5	0.3	22.3
*Il Corriere della Sera*	1.5	75.1	0.6	22.7
Other newspapers^a^	1.1	74.0	0.1	24.8
Rai (Google News)	0.5	49.3	0.0	50.2
Mediaset (Google News)	0.0	38.6	1.3	60.1

^a^This item includes *Il Sole 24 Ore*, *Il Fatto Quotidiano*, *Il Giornale*, and *La Stampa*.

### Web Users’ Interest in Mass Media Infodemic Videos

The YouTube channels of the following news broadcasts were investigated: Rai (4.08 million subscribers), LA7 Attualità (730,000 subscribers), MediasetPlay (605,000 subscribers), *La Repubblica* (576,000 subscribers), *Corriere della Sera* (145,000 subscribers), and Tgcom24 (52,700 subscribers). Only videos with over 100,000 views were considered for analysis. The views of videos containing the keyword *coronavirus* were substantially higher than those of videos containing the keywords *COVID-related* and *sars-cov-2* (50.07 million views vs 8.51 million views, δ%=141.9). Moreover, some of the videos with the most views had extremely infodemic titles, such as the following Italian headlines, here provided as their English translations: “Covid does not exist” (621,945, first on La7 Attualità), “The 2015 Rai-Leonardo video on the virus created in China in the laboratory. The scientific community...” (401,648, fourth on *Il Corriere della Sera*, “Coronavirus, Vittorio Sgarbi: ‘It is not an epidemic because there is no risk of death’” (440,296, second on La7 Attualità), “Coronavirus, Sgarbi: The confinement of asymptomatic people is fascism” (396,144, sixth on *Il Corriere della Sera*), “Coronavirus, the Senate conference of ‘deniers’: In Italy, the virus no longer exists” (189,655, 47th on *La Repubblica*), “Covid, Prof. Roberto Bernabei: It’s a normal disease...” (141,233, 21st on La7 Attualità), “Coronavirus, the reassurance of the infectious disease specialist Matteo Bassetti: It’s not an infection...” (the video discusses the similarities between COVID-19 and common seasonal influenza; 203,347, 14th on La7 Attualità). Finally, the presence of growing interest in information channels that disclose serious fake news must be taken into account. In particular, the YouTube channel ByoBlu (524,000 subscribers) often shares scientifically unjustified opinions of people who have become famous for their conspiratorial positions on COVID-19. However, an exact estimate of the extent of this phenomenon is difficult to make, as many videos have been blacked out by YouTube due to their misinformative nature [53].

### Web Users’ Interest in Mass Media Infodemic Statements

**Figure 4:**
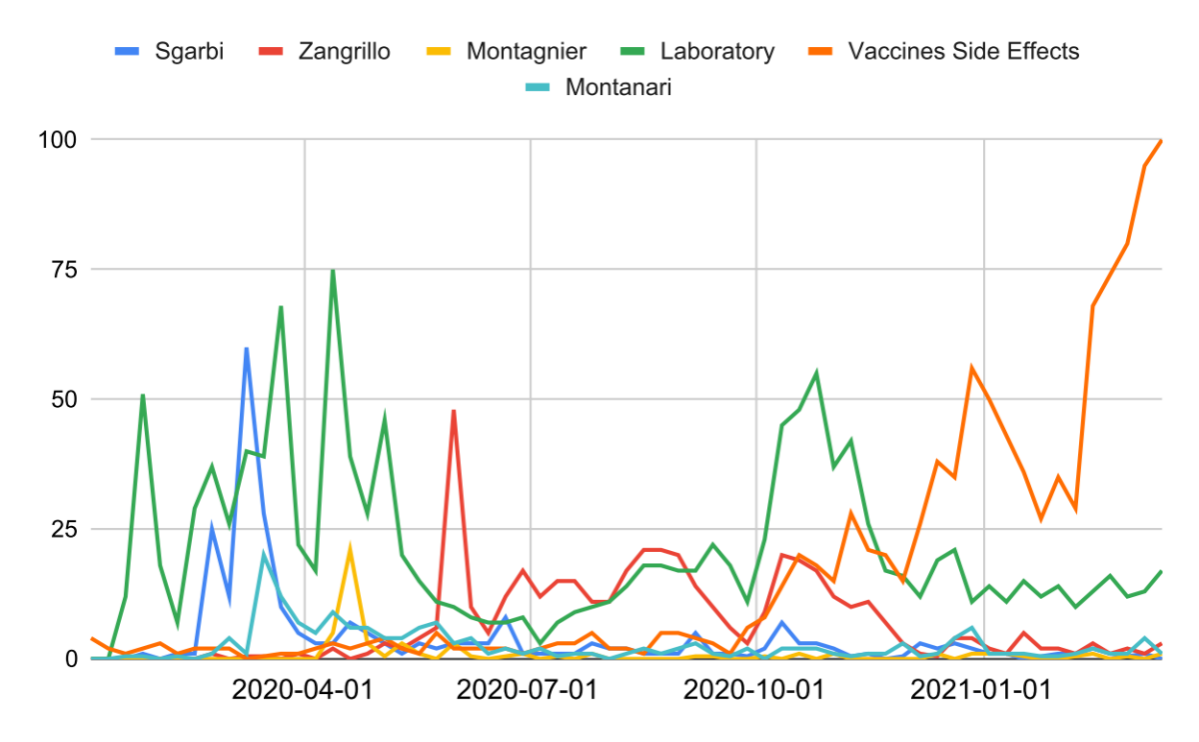
Relative search volumes (RSVs) of infodemic queries since the start of the COVID-19 pandemic. The *astrazeneca* query is not shown to enable visualization of the other RSV trends.

Some statements from prominent personalities, including scientists, have directed the web interest of users toward disinformation and misinformation (Figure 4).

Among these statements, on February 23, 2020, Dr Maria Rita Gismondo compared COVID-19 to seasonal influenza [54]. On the same day, the RSV of the *coronavirus flu + covid flu* query increased from 0 to 100, maintaining high values until March 22 (mean RSV=40.2, SEM 3.2). On February 24, 2020, politician Vittorio Sgarbi minimized the risk of death from COVID-19 without any supporting scientific evidence [55]. On the same day, the proper RSV of the *coronavirus sgarbi* + *covid sgarbi* query increased from 2 to 75. Moreover, Sgarbi became the protagonist of a long series of infodemic statements, including incitement to violate the anti–COVID-19 regulations [56]. Over the same period, the RSV from the previous query remained high, achieving two new maxima on March 10 and 14 (RSV=100 and 93, respectively). Other major fake news stories that circulated through the media involved the creation of COVID-19 in a Chinese laboratory. This phenomenon was supported by a 2015 report of a local news program, repurposed by the media; the words of the politician Matteo Salvini; and the statements of the Nobel Prize winner Luc Montagnier [10,57]. Another promoter of conspiracy theories was Dr Stefano Montanari [58]. Soon after his statements, there was a spike in the RSV of the query *montanari coronavirus* + *montanari covid*. On May 31, 2020, Dr Alberto Zangrillo declared the disappearance of COVID-19 [59]. The same day, there was a heavy rise in the query *coronavirus zangrillo* + *covid zangrillo*. However, the major infodemic impact of the media has been on vaccines, especially for the AstraZeneca vaccine. Figure 4 shows a clear trend in the weekly RSV of the query *vaccines side effects* from the second week of February 2021 onward (SS=10, Mann-Kendall *P*=.009). By narrowing the range to obtain daily RSVs, a clear level shift can be observed from February 12 to 14, 2021. In particular, comparing the period of January 20 to February 13 with that of February 17 to March 9, a dramatic percentage increase in web interest (∆%=205.7, *t*=17.4) was observed. However, the peak was reached on March 11 (RSV=100), with a sharp rise starting on March 9. In the same period, a long series of misleading headlines were published by the main Italian newspapers, thus fomenting the distrust of vaccines [60,61]. Finally, in the same period, the RSV of the query *astrazeneca side effects* showed an extreme increase (∆%=2991.4, *t*=16.9).

## Discussion

### Principal Findings

The results of this paper suggest that the main Italian mass media outlets have not only heavily influenced the trend of web interest in COVID-19 but also had a strong impact on the terms adopted by users to identify the novel coronavirus. In particular, although official sources such as the Ministry of Health and PubMed mainly adopted the scientific denominations (eg, 2019-nCoV, COVID-19, novel coronavirus), the principal Italian newspapers and national information channels, such as *La Repubblica*, *Il Corriere della Sera*, Rai, and Mediaset, have given resonance to more infodemic names. However, the use of these terms was neither constant nor homogeneous during the pandemic: indeed, from January to March 2020, the use by the media of monikers such as “coronavirus” and “Chinese coronavirus” reached worrying values ​​(over 90% and 6%, respectively). From April to May 2020, the use of terms such as “COVID” and “COVID-19” became common, although the generic “coronavirus” remained the most popular. From May 2020 to date, COVID-19–related names have become more common (except for Rai and Mediaset, where “coronavirus” is still widely adopted). In this scenario, web searches faithfully followed the news published on COVID-19. Especially during the beginning of 2020, all RSV peaks were associated with outbreaks or lockdown announcements. Moreover, marked and significant correlations were found between newspaper headlines and RSVs. Because similar correlations have not been highlighted with other potentially associable quantities, such as COVID-19 statistics (eg, new cases, new deaths), the terms most adopted by users mirror those used by the press, and numerous publications have previously shown mass media influences on web searches, we conclude that the press played a substantial role in creating a mass culture on COVID-19. Furthermore, our findings show that the terms used by mass media to identify the virus in the very early stages definitely affected users’ vocabulary and were difficult to replace. For example, although the scientific name “COVID-19” was introduced on February 11, 2020, by the World Health Organization and became predominant in major national newspapers in May 2020 [62], Italian netizens equalized it with the use of the name “coronavirus” only in September 2020. Alongside this, web users have shown a strong interest in videos, articles, and services shared by mass media supporting conspiracy theories, fake news, and unjustified claims. Specifically, the authors of this paper express concern that this material has reached millions of views and is very dangerous for public health and safety.

### Comparison With Recent Literature

To the best of the authors’ knowledge, this is the first study to propose a longitudinal analysis on the relationship between web RSVs and the headlines of stories in the main Italian media during the COVID-19 pandemic. Nonetheless, our results are supported by other recent literature. Regarding R1, Su et al [21] criticized the media for having done little to limit the spread of the infodemic during the current global health crisis, stressing the need to improve the communication system. In particular, they harshly criticized the multitude of journalists who, despite the required ethical and professional standards, adopted stigmatizing monikers linked to ethnicities or geographic locations, such as “Chinese virus,” “Wuhan virus,” and “China virus.” Wen et al [63] also denounced the use of headlines such as “Chinese virus pandemonium.” Indeed, the mass media have strict responsibilities for spreading these inappropriate names, which have fueled xenophobia toward the Chinese population and conspiracy theories globally [9,10,63-66]. These considerations acquire greater relevance considering R2: specifically, other studies have highlighted a strong influence of the media on web searches and interests. For example, Szmuda et al [67] showed that Google Trends is better at analyzing COVID-19–related media clamor than actual disease incidence. Tejedor et al [68] examined the front pages of Italian and Spanish newspapers during COVID-19 (including *Corriere della Sera* and *La Repubblica*), emphasizing that “they still play a crucial role in molding public opinion by offering more interpretative content.” The same authors stressed the need to increase information literacy in the population and media professionals “to achieve rigorous and responsible health information.” Huynh Dagher et al [27] showed that even the web interest in cutaneous COVID-19 symptoms was strongly influenced by media coverage and government policies in France, Spain, Italy, and the United States, calling for caution in adopting Google Trends as an epidemiological monitoring tool. In contrast, only weak correlations were found in Germany and the United Kingdom. Ergo, the media impact may be dependent on the country. Likewise, Fernández-Torres et al [69] pointed out that although Spanish citizens are interested in news about the novel coronavirus, the mass media often provide low-quality information. Finally, concerning R3, newspapers, television stations, and news channels have given too much resonance to conspiratorial opinions and uninformed or incompetent people [9,10]. The most striking case is that of the former president of the United States, Donald Trump, who gave extremely dangerous advice on how to fight SARS-CoV-2, such as consuming unapproved drugs or injecting disinfectant [10]. Furthermore, Trump repeatedly accused China of deliberately creating the virus and adopted a wide variety of racist monikers [70]. A second equally notable case is that of Luc Montagnier, winner of a Nobel Prize in Medicine, who claimed, without any supporting scientific evidence, that SARS-CoV-2 was the result of laboratory manipulation of HIV [10]. In this regard, Moscadelli et al [71] reported an increase in web searches for coronavirus laboratory–related keywords in conjunction with Montagnier’s statement and pushed for greater control of information shared by the media. Ferreira et al [72] established a strong dependence of Portuguese citizens on conventional mass media during the COVID-19 pandemic, underlining that most users have shown greater trust in these media outlets than in social media. Similar findings were found by Fernández-Torres et al [69] for Spain. However, the authors of both papers agree that citizens believe that news and newspapers are not free from false information. This evidence cannot be judged in positive or negative terms because it is not known which types of information are cataloged as such; for example, some Italian conspiracists believe that journalists are purposely exaggerating the number of COVID-19 deaths to instill fear and acquiescence. Nonetheless, this phenomenon testifies to the climate of distrust that afflicts the population and the need to create sources that are perceived as reliable.

### Practical Implications

This study provides evidence that in Italy, the mass media have strongly influenced web users in the adoption of names to identify COVID-19 and favored the diffusion of infodemic monikers (R1); the mass media have determined the trend of web searches on COVID-19 (R2); and the web interest in infodemic news shared by mass media has been high (R3). The use of too-generic terms by the mass media is plausibly an attempt to simplify information for the public. This would justify the easy success of names such as “coronavirus” instead of “novel coronavirus 2019” or “covid” instead of “COVID-19”. Because Italy is one of the nations in the world with the highest percentage of functionally illiterate people [73], using simple and immediately understandable words is a fundamental aspect of health disclosure [74]. In this regard, the first name, 2019-nCoV, may have been too technical to be adopted by citizens. Because the initial denomination of a disease affects the behavior of users over a long period, we suggest that more attention should be paid when making a definitive choice. Alongside this, the use of racist monikers was due both to the naive attempt to contextualize the virus in the geographical scenario and the politicization of COVID-19 [75]. These monikers were often contained in headlines, which can bias the entire reading of an article [76]. For these reasons, we suggest that government authorities impose more controls and restrictions on newspaper headlines and expect greater discretion and sensitivity from the editors of news organizations.

### Limitations

This analysis was subject to some limitations. First, there are no guarantees that the interest of Italian web users can represent a true-to-life picture of the entire Italian population’s interests; thus, the conclusions of this paper are limited to Italian internet users. Second, causal correlations were only searched between Google Trends RSVs and the headlines used by the two most-read Italian newspapers. Future research could investigate the correlations between the Italian population and the mass media, also involving non–internet users and all Italian media. Thirdly, RSVs provide information about the relative number of web searches on COVID-19 but not their exact number. However, as previous literature has shown that the numbers of COVID-19–related web searches and discussions have always been high [22], related trends also provide valuable information. Finally, the paper cannot represent the totality of the interests of web users that arose during the COVID-19 pandemic.

### Conclusions

Because the leading Italian mass media have strongly influenced both the risk perception and interest of Italian web users toward the novel coronavirus (SARS-CoV-2, which causes COVID-19), we suggest that the Italian authorities place strict and effective controls on the information circulating in Italy. Furthermore, the authors of this paper recommend that the directors of news channels and newspapers adhere to the official name of COVID-19 (as well as any other virus or disease), favoring a less sensationalistic disclosure policy. Finally, the authors of this paper recommend carefully weighing the influence of mass media on users’ web searches before adopting any epidemiological predictive models based on Google Trends or similar infoveillance tools.

## Appendix

Multimedia Appendix 1 Trend of COVID-19 symptoms–related web queries from 2017 to 2021 (Google Trends). The relative search volumes shown are cumulative monthly.

## Abbreviations

C19: COVID-19 cases and deaths
I-scale: infodemic scale
RSV: relative search volume
SEM: standard error of the mean
SS: Sen slope
