# Supplementary material for: Influence of Mass Media on Italian Web Users During the COVID-19 Pandemic: Infodemiological Analysis
Source: JMIRx Med. 2021 Oct 18;2(4):e32233. doi: 10.2196/32233 (PMC8601032; doi:10.2196/32233)
Supplement: Multimedia Appendix 1 [file xmed_v2i4e32233_app1.docx]

***Multimedia Appendix 1***

**Correlations between *RSVs* and *newspaper headlines***

Correlations between “coronavirus” *RSV* and *La Repubblica headlines* from January 19 to March August 8, 2020: *r1=.95, P1<.001; r2=.78, P2<.001; R3=.93, P3=.008; R4=.94, P4<.001; R5=.94, P5<.001; r6=.97, P6<.001*.

Correlations between “coronavirus” *RSV* and *Il Corriere della Sera headlines* from January 19 to March 13, 2020: *r1=.61, P1=.037; r2=.56, P2=.013; R3=.91, P3=.012; R4=.24, P4=.604; R5=.57, P5=.067; r6=.96, P6<.001*.

**COVID-19 symptoms-related web searches**

As for the queries previously investigated, the keyword “coronavirus symptoms + covid symptoms” in the timelapse January 20 – May 23 has been influenced by media (*ρ=.92±.03,∀P<.007*). Its RSV was highly sensitive to news, reaching peaks of on January 31, February 23, and March 9. Among the COVID-19 symptoms reported by the Italian Ministry of Health, the most typed on search engines during the whole pandemic were the Italian terms for fever (*RSV*=49±4.6*), cough (*23.0±3.5*), cold (*15.2±2.2*), sore throat (*12.5±1.3*), and pneumonia (*12.3±3.2*). During the previous 3 years (2017-2019), these showed winter seasonalities but neither global nor local trends or unit-roots (*∀ADF P<.004,∀m<0.05*, Supplementary Figure 1). This allowed us to easily identify two anomalies: February – May 2020, with *∆_peak_%∈[50,835], t∈[14,21]*, and August – November 2020, with *∆_peak_%∈[46,264], t∈[2.8,6.2]* (except for “cough”). Therefore, the impact of the pandemic on this type of search has been substantial. Nonetheless, there is evidence that counteracts the potential use of such web searches to predict COVID-19 cases: in fact, the general *RSV* trend in the period February – May 2020 presents more similarities and correlations with *newspaper titles* than with *COVID-19 cases* (Supplementary Figure 2).


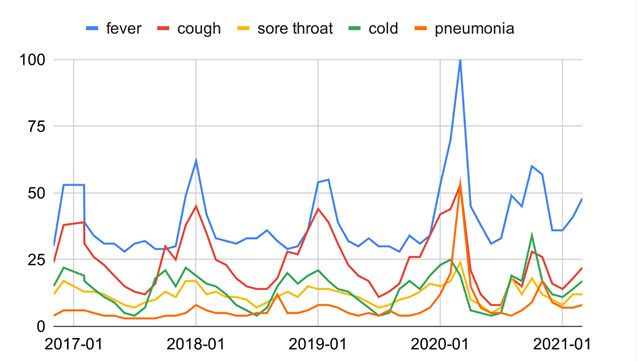
**Supplementary Figure 1.** Trend of COVID-19 symptoms-related web queries from 2017 to 2021 (Google Trends). The relative search volumes shown are cumulative monthly.


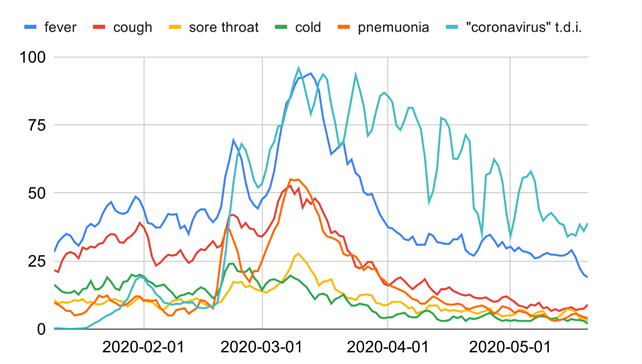
**Supplementary Figure 2.** Comparison between COVID-19 symptoms-related web queries (Google Trends relative search volumes) and “La Repubblica” newspaper titles containing the word “coronavirus” from January to May 2020.

In particular, although the analysis of cross-correlations has identified some optimum values with a lag of 2 days, the onset of local RSV trends never preceded that of newspaper headlines. Additionally, we once again observed two clear decreasing trends in RSV between late February and early March and after 15 March, despite the continued increase in COVID-19 cases.
